# Characteristics of HBV Novel Serum Markers across Distinct Phases in Treatment-Naïve Chronic HBV-Infected Patients

**DOI:** 10.1155/2022/4133283

**Published:** 2022-07-14

**Authors:** Hao Liao, Le Li, Wei V. Zheng, Jun Zou, Guangxin Yu, Lanlan Si, Feilin Ge, Tao Zhou, Dong Ji, Xiangmei Chen, Dongping Xu, Guanxun Cheng, Yan Liu, Junhui Chen

**Affiliations:** ^1^Intervention and Cell Therapy Center, Peking University Shenzhen Hospital, Shenzhen Peking University-The Hong Kong University of Science and Technology Medical Center, Shenzhen, 518035 Guangdong Province, China; ^2^Department of Infectious Diseases, The Fifth Medical Center of Chinese PLA General Hospital, Beijing 100039, China; ^3^Department of Microbiology & Infectious Disease Center, School of Basic Medical Sciences, Peking University Health Science Center, Beijing 100191, China

## Abstract

**Methods:**

A total of 111 patients in total from different disease phases were recruited, including 21 in immune-tolerant (IT) phase, 49 in immune-clearance (IC) phases, 29 in immune-control or low replicative (LR) phase, and 12 in reactivation phases. Serum HBV RNA, anti-HBc, HBcrAg, and intrahepatic covalently closed circular DNA (cccDNA) were quantified and each of these indicator's correlation with liver inflammation was analyzed.

**Results:**

HBeAg-positive individuals had significant higher serum levels of HBV RNA and HBcrAg than those who were HBeAg negative, similar to that of serum HBV DNA. Comparatively, HBV RNA (*r* =0.79, *P* < 0.01) and HBcrAg (*r* =0.78, *P* < 0.01) had almost same higher overall correlation with the cccDNA, as that of HBV DNA (*r* =0.81, *P* < 0.01). Serum anti-HBc level (*r* = -0.52, *P* < 0.05) is negatively correlated with cccDNA level at IT phase rather than the other three phases. When set the cutoff value at 4.00 log_10_ IU/mL, serum anti-HBc showed potential to indicate liver inflammation, with AUC as 0.79 and the specificities as 78.85% for HBeAg positive, and with AUC as 0.72 and the specificities as 62.16% for HBeAg-negative patients, respectively.

**Conclusions:**

In treatment-naïve patients, levels of serological markers HBV RNA and HBcrAg could mirror intrahepatic cccDNA level, but were not superior to HBV DNA level. Serum anti-HBc level had certain potential to be used as a predicting marker for liver inflammation.

## 1. Introduction

An estimated 257-291million people worldwide are chronically infected with hepatitis B virus (HBV), and the interaction between HBV replication and the host immune response may lead to a variety of clinical complications from inflammation to liver cirrhosis and hepatocellular carcinoma [1]. The natural history of chronic HBV infection has been divided into four or five phases, according to the American Association for the Study of Liver Diseases (AASLD) or European Association for the Study of the Liver (EASL) [2, 3]. AASLD suggested the four phases of natural history (immune-tolerant phase, HBeAg-positive immune-active phase, inactive CHB phase, and HBeAg-negative immune reactivation phase); each phase has distinct characteristics on the HBeAg, HBV DNA levels, alanine aminotransferase (ALT) values, and grades of liver inflammation.

Not all patients with chronic HBV infection have chronic hepatitis (CHB), and monitoring viral replication is important for the management of HBV. At the same time, high levels of replication do not necessarily predict high levels of inflammation, so indicators of viral replication (HBV DNA or covalently closed circular DNA (cccDNA)), serological markers, and liver inflammation should all be considered in determining whether and when to initiate treatment. The cccDNA, as the template of HBV transcription, contributes to infection persistence [4, 5]. Recently, novel serum surrogate makers of cccDNA including serum hepatitis B core-related antigen (HBcrAg) and HBV RNA have attracted research interest [6]. Our previous study, as well as work by others, have demonstrated that serum HBV RNA from the pgRNA are not or partially reverse transcribed in encapsidated particles [7], which may reflect cccDNA transcriptional activity [8, 9]. Serum HBV RNA may be an early clinical indicator of HBeAg seroconversion, virological response, and HBsAg reversion during pegylated interferon-*α* or nucleoside/nucleotide analogues (NAs) antiviral therapy [10–12]. Serum HBcrAg has an excellent correlation with cccDNA transcription in CHB patients and is also suggested as a serum marker for cccDNA [13]. In addition, HBcrAg shows high diagnostic performance in the accurate identification of patients with HBeAg-negative CHB [14]. When serum HBV DNA level is too low to be detected, monitoring HBV RNA and HBcrAg can be used to reflect viral replication in NAs-treated patients [15]. In addition, it has been reported that serum hepatitis B core antibody (anti-HBc) levels in CHB patients are useful markers of NAs and pegylated interferon therapy efficacy [16, 17]. More importantly, serum anti-HBc is part of the host HBV-specific adaptive immune component, and elevated anti-HBc levels in CHB patients with normal alanine aminotransferase indicate liver inflammation [18, 19].

Although the clinical characteristics of HBcrAg, HBV RNA, and anti-HBc have been studied before, they had not been studied in one cohort of patients. In this study, we provided a comprehensive picture of all these serological markers and indicators of viral replication in relation to liver inflammation in untreated patients across the distinct phases of HBV chronic infection, with an emphasis on the serum anti-HBc.

## 2. Methods

### 2.1. Patients and Study Design

The study included a cross-sectional set of 111 treatment-naïve CHB patients, of whom 21 (18.92%) were in the immune-tolerant (IT) phase, 49 (44.14%) in the immune-clearance (IC) phase, 29 (26.13%) in the low replicative (LR) phase, and 12 (10.81%) in the reactivation phase. Disease phases were categorized referring to the Asian-Pacific clinical practice guidelines on the management of hepatitis B: a 2015 update [20]. All patients were recruited from the Fifth Medical Center of PLA General Hospital from January 2017 to December 2019. Only patients with liver biopsy were included. There was no evidence for concomitant HCV, HDV, or HIV infection or alcoholic liver disease, autoimmune liver disease. Patients with cirrhosis and hepatocellular carcinoma were excluded. Fibrosis and necro-inflammatory activity were quantified by experienced histopathologists using the METAVIR criteria [21]. At the time of liver biopsy, serum samples were collected and stored at -80°C until use. The Ethics Committee of the Fifth Medical Center of PLA General Hospital approved the study protocol. The enrolled patients met the requirement for inclusion in the database of the Fifth Medical Center of PLA General Hospital. Written informed consent was obtained from all patients.

### 2.2. Determination of HBV DNA and Serological HBV Markers

HBeAg and HBsAg levels were determined using a Roche Cobas e601 analyzer (Roche Diagnostics Ltd., Burgess Hill, UK) and expressed according to a cutoff index. HBsAg quantification was performed using Roche Cobas e601 (Roche Diagnostics) with a 0.05–250 IU/mL linear detection range for HBsAg. Real-time fluorescent quantitative PCR was used to quantify serum HBV DNA, and the method has a lower limit of quantitation (LLOQ) of 20 IU/mL.

### 2.3. Quantitation of Serum Anti-HBc, HBV RNA, and HBcrAg

Serum anti-HBc was quantified on the Caris 200 Platform following the kit manufacturer's instructions (Wantai, Beijing, China) using a recently developed double-sandwich immunoassay method. Calibration standards of the assay used standards provided by the World Health Organization. The assay has a LLOQ of 0.25 IU/mL and a linear detection range of 2-5 log_10_ IU/mL.

After serum HBV RNA was extracted, one-step real-time RT-PCR amplification was performed according to the kit manufacturer's instructions (Beijing Hotgen Biotech Co., Ltd., Beijing, China). The assay has a LLOQ of 100 copies/mL and a linear detection range of 3-8 log_10_ copies/mL. Values below the LLOQ were defined as half of constant LLOQ, i.e., 50 copies/mL in statistical analysis.

Serum HBcrAg levels were determined using a chemiluminescent enzyme immunoassay according to the kit manufacturer's instructions (Lumipulse System, Fujirebio Inc., Tokyo, Japan). The assay has a LLOQ of 1,000 U/mL and a linear detection range of 3-7 log_10_ U/mL. Samples with a value beyond detection limit were retested at a 1 : 100 dilution. Values below the LLOQ (not quantifiable but detectable) were still recorded and analyzed.

### 2.4. Quantitation of Intrahepatic HBV Total DNA and cccDNA

Extraction and quantification of intrahepatic HBV cccDNA were performed as we previously described [22]. First, the intracellular free-microchromosomal DNA and genomic DNA were extracted by the QIAamp DNA Mini Kit according to the manufacturer's instructions (QIAGEN, GmbH, Hilden, Germany). PSAD was applied to digest HBV dsDNA, rcDNA, and ssDNA (Epicentre, Madison, WI, USA). Then, cccDNA-selective amplification was performed using Rolling Circle Amplification (RCA) method with Phi29 DNA polymerase (New England Biolabs, Worcester, MA). The RCA products were subjected to further amplification and quantification using TaqMan real-time PCR mediated by probes targeting gap regions of the viral genome and a cccDNA-selective primer pair. PSAD and phi29 DNA polymerase treatment was not required for detection of HBV total DNA. Number of cells was calculated according to primers and probes for a reference control DNA fragment for human beta-actin. The LLOQ for this assay was 0.01 copies/cell.

### 2.5. Assessment of Liver Inflammation

METAVIR scoring is used for histopathological assessment of the extent of inflammation and fibrosis in a liver biopsy of patients [21]. The grade indicates the activity or degree of inflammation and the stage indicates the amount of fibrosis. Here we collected the grade for the inflammation activity. Namely, G0: no activity G1: mild activity; G2: moderate activity; G3: severe activity. Patients of the four phases were regrouped into two groups according to their histological inflammation status: the no to mild degree group (Metavir score G0-G1) and the moderate to severe group (Metavir score G2-G3).

### 2.6. Statistics Analysis

Medians and interquartile ranges are presented for the results. The *χ*^2^ test and the Mann–Whitney *U* test were carried out for categorical measures and continuous measures in univariate analysis. Correlation between two parameters was analyzed using Spearman's method. The diagnostic validity of variables was defined as the area under the receiver-operating characteristic (AUC) curve, and the difference was tested by McNeil and Hanley. IBM SPSS (version 21) was used for all statistical analyses. GraphPad Prism (version 8.0) was the software used for graphical analyses and data presentation A two-tailed *P* value <0.05 was considered statistically significant.

## 3. Results

### 3.1. Clinical Characteristics of Patients

This study recruited a total of 111 antiviral treatment naïve individuals with chronic HBV infection at distinct disease progression phases, with a median age of 35 (29, 42) yrs, and 61.26% (68/111) were male (Table 1). Among whom, 70 (63.06%) were HBeAg positive and 41 (36.94%) were HBeAg negative. Significantly elevated ALT (35 U/L is upper limit of ALT normal) and aspartate aminotransferase (AST, 40 U/L is upper limit of AST normal) were observed in the IC- or reactivation-phase patients, concordant to the new EASL guideline for the management of CHB [2], in which patients in the IC or reactivation phase were identified as HBeAg-positive or HBeAg-negative CHB patients. As expected, the overall HBV DNA level in HBeAg-negative patients 2.93 (2.24, 3.34) log_10_ IU/mL was significantly lower than that of HBeAg-positive patients 8.55 (7.85, 9.00) log_10_ IU/mL (*P* < 0.001). Noticeably, the serum HBV DNA level in the reactivation phase 4.27 (3.44, 5.08) log_10_ IU/mL was significantly increased as compared to that of patients in LR phase 2.37 (1.94, 3.04) log_10_ IU/mL (*P* < 0.001). Meanwhile, the HBsAg level in HBeAg-positive patients decreased significantly, forming a median of 52010 (36221, 77630) IU/mL in the IT phase to 27173 (5731, 49565) IU/mL in the IC phase (*P* = 0.009), which were significantly higher than that of HBeAg-negative patients; the medians of HBsAg were 1516 (231, 4814) IU/mL in the LR phase and 1176 (608, 7741) IU/mL in the reactivation phase.

### 3.2. Serum HBV RNA, HBcrAg, Anti-HBc, and Intrahepatic cccDNA Levels across Distinct Disease Phases

**Figure 1:**
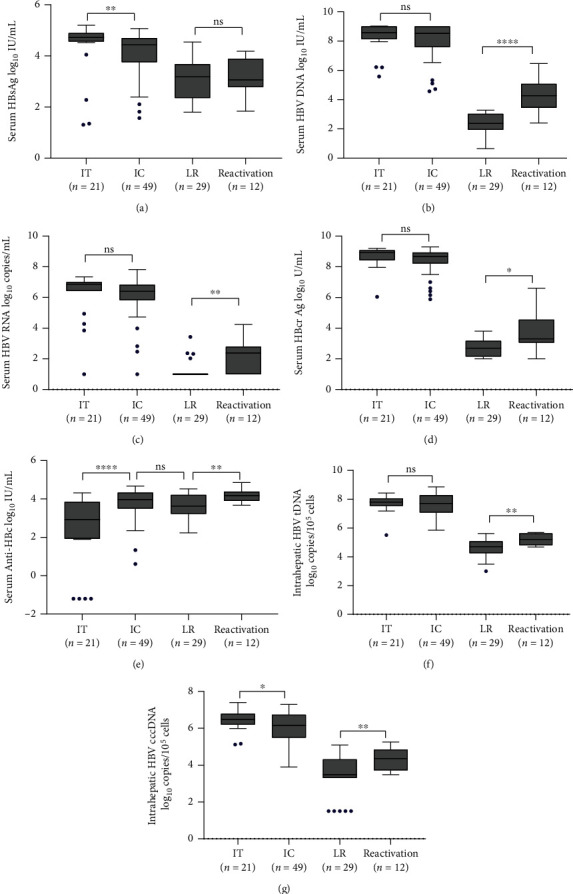
HBV serum markers and intrahepatic cccDNA levels across the distinct phases of chronic HBV infection. Abbreviations: IT: immune-tolerant phase; IC: immune-clearance phases; LR: low-replicated phase; HBV: hepatitis B virus; HBsAg: hepatitis B surface antigen; HBcrAg: hepatitis B core-related antigen; anti-HBc: hepatitis B core antibody; tDNA: total DNA; cccDNA: covalently closed circular DNA; n: number of cases; ∗∗∗∗*P* < 0.0001; ∗∗∗0.0001 < *P* < 0.001; ∗∗0.001 < *P* < 0.01; ∗0.01 < *P* < 0.05; ns, *P* > 0.05.

As shown in Figure 1, similar to serum HBV DNA levels, serum HBV RNA levels in the IT-phase and IC-phase patients [6.87 (6.42, 7.00) log_10_ copies/m, 6.40 (5.83, 6.83) log_10_ copies/mL] were significantly higher than those in the LR-phase and reactivation-phase patients [below the LLOQ, 2.39 (1.00, 2.80) log_10_ copies/mL]. The changes in intrahepatic HBV tDNA and cccDNA levels shared same trend across four disease phases. The cccDNA levels were 6.48 (6.20, 6.79), 6.16 (5.49, 6.74), 3.48 (3.30, 4.32), and 4.36 (3.72, 4.85) log_10_ copies/10^5^cells for the IT-, IC-, LR-, and reactivation-phased patients, respectively. In HBeAg-positive patients, IT-phase patients had higher serum HBcrAg levels than IC-phase patients, and the difference was marginally significant [8.91(8.42, 9.07) *vs.* 8.66 (8.21, 8.93) log_10_ U/mL, *P* = 0.055], while in HBeAg-negative patients, LR-phase patients had lower HBcrAg levels than reactivation-phase patients [3.31 (3.05, 4.56) *vs.* 2.70 (2.15, 3.18) log_10_ U/mL, *P* = 0.012]. During the reactivation phase, serum HBV DNA, HBV RNA, and liver HBV cccDNA levels were all re-increased.

Serum anti-HBc level in the IT phase was the lowest compared with the other three phases, [i.e., 2.93 (1.93, 3.85) vs. 3.97 (3.50, 4.34), 3.63 (3.22, 4.21), and 4.12 (3.90, 4.38) log_10_ IU/mL]. Notably, the pattern of changes in anti-HBc levels across the four disease phases was different from those of HBV RNA, HBV DNA, and HBcrAg levels, with significant increases from IT to IC, and LR to reactivation phase.

### 3.3. The Correlation between Intrahepatic cccDNA and Serum HBV Markers

**Figure 2:**
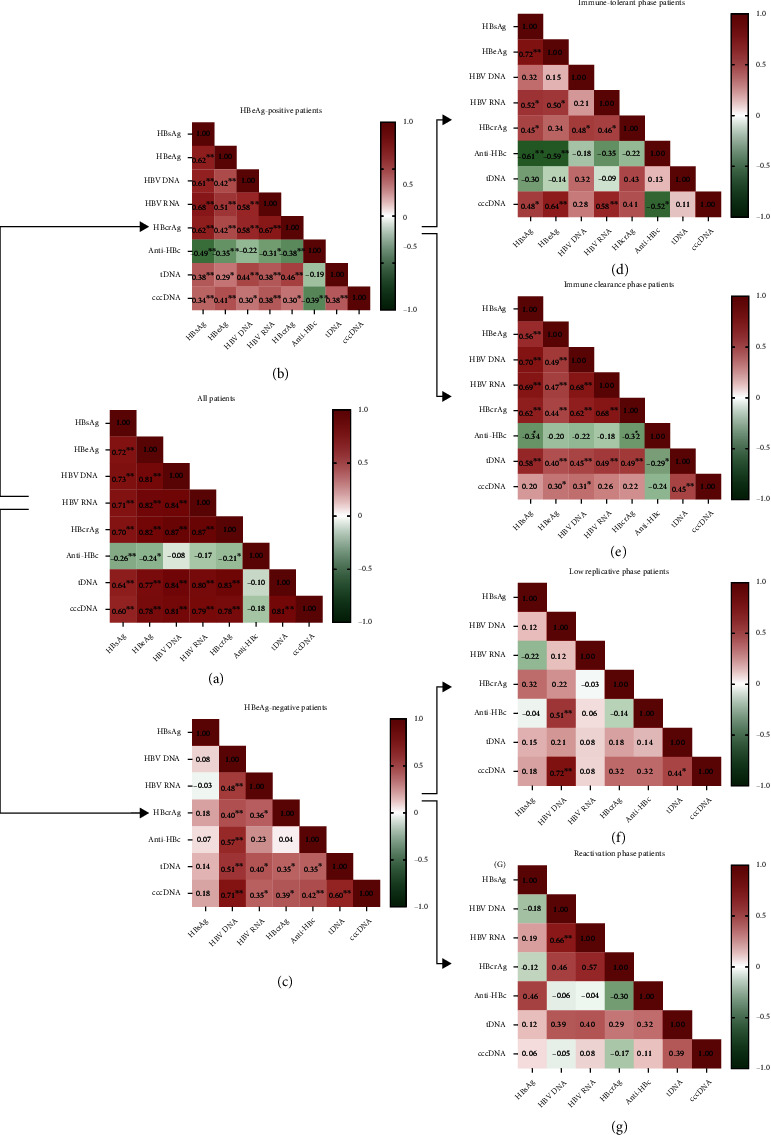
The correlation analysis of HBV markers across the distinct phases. The correlations of each serum HBV markers in the overall patients (a), HBeAg-positive patients (b), HBeAg-negative patients (c), IT-phased patients (d), IC-phased patients (e), LR-phased patients (f), and reactivation-phased patients (g). The value in the cell was Spearman's correlation coefficient (*r*). The bar of white-green-red indicates a growing correlation. Abbreviations: cccDNA: covalently closed circular DNA; HBV: hepatitis B virus; HBeAg: hepatitis B e antigen; HBsAg: hepatitis B surface antigen; HBcrAg: hepatitis B core-related antigen; anti-HBc: hepatitis B core antibody; tDNA: total DNA.

Next, Spearman's correlation coefficients (*r*) analysis was conducted to assay the correlation of viral marker with cccDNA. As expected, intrahepatic HBV tDNA and cccDNA showed a synchronous correlation trend. Except for anti-HBc, strong correlations were observed between intrahepatic HBV cccDNA level and the HBV serological markers in all patients, in an order from strong to weak as HBV DNA (*r* =0.81, *P* < 0.01) > HBV RNA (*r* =0.79, *P* < 0.01) ≈ HBcrAg (*r* =0.78, *P* < 0.01) > HBeAg (*r* =0.78, *P* < 0.01) > HBsAg (*r* =0.60, *P* < 0.01) (Figure 2(a)). Stratified analysis revealed that in HBeAg-positive patients, the correlation between intrahepatic cccDNA and serum HBV DNA (*r* =0.30, *P* < 0.05), HBcrAg (*r* =0.30, *P* < 0.05), HBV RNA (*r* =0.38, *P* < 0.01), HBeAg (*r* =0.41, *P* < 0.01), and HBsAg (*r* =0.34, *P* < 0.01) was similar (Figure 2(b)), while in HBeAg-negative patients, serum HBV DNA showed a strongly correlation with intrahepatic cccDNA (*r* =0.71, *P* < 0.01), followed by serum HBcrAg (*r* =0.39, *P* < 0.05), HBV RNA (*r* =0.35, *P* < 0.05), and no correlation for HBsAg (*r* =0.18, *P* > 0.05) (Figure 2(c)). As a consequence, the correlations between HBsAg and the other HBV markers were poor. However, a much stronger correlation between serum HBV DNA with intrahepatic cccDNA was noticed in those HBeAg-negative patients (*r* =0.71, *P* > 0.01), though in general the other serum HBV markers had weak correlations with the intrahepatic cccDNA in such patients. Interestingly, stratification analysis showed that anti-HBc was negatively correlated with intrahepatic cccDNA in HBeAg-positive patients (*r* = -0.387, *P* < 0.05), while in HBeAg-negative patients, the correlation was positive (*r* =0.419, *P* < 0.05).

In HBeAg-positive patients, the correlation of serum HBcrAg with serum HBV DNA and RNA was 0.48 (*P* < 0.01) and 0.46 (*P* < 0.01) in IT phase, and 0.62 (*P* < 0.01) and 0.68 (*P* < 0.01) in IC phase (HBeAg-positive hepatitis), respectively (Figures 2(d) and 2(e)). However, such correlations were only seen in HBeAg-negative patients in general (*r* =0.51, *P* < 0.01 for DNA; *r* =0.40, *P* < 0.05 for RNA, respectively), but neither in low replicative nor in reactivation phases, even though without the interfering of HBeAg on HBcrAg measurement, perhaps due to the too small number of patients (Figures 2(f) and 2(g)). On the other hand, the correlation between serum HBV RNA and serum HBV DNA was only seen in patients of IC (HBeAg-positive hepatitis) (*r* =0.68, *P* < 0.01) and reactivation (HBeAg-negative hepatitis) (*r* =0.66, *P* < 0.01) phases, but neither in the HBeAg-positive IT nor in the HBeAg-negative lower replication phases. These result to some extent, different from previous reports.

### 3.4. The cccDNA Transcription and pgRNA Reverse Transcription Efficiencies across Distinct Disease Phases

**Figure 3:**
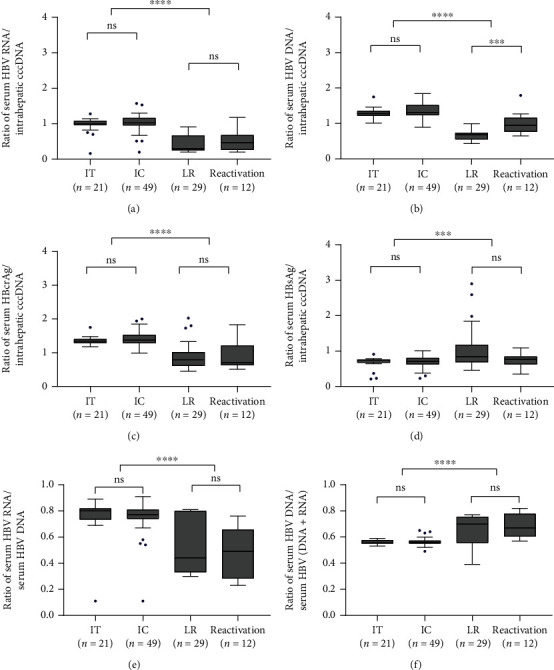
The transcriptional efficiency of cccDNA and the reverse transcription efficiency of pgRNA across distinct disease phases. The transcriptional efficiency of cccDNA was determined by the ratio of serum HBV RNA (a), HBV DNA (b), HBcrAg (c), and HBsAg (d) to intrahepatic cccDNA. The reverse transcription efficiency of HBV pgRNA represented as a ratio of serum HBV RNA to HBV DNA was inversely related (e). The ratio of serum HBV DNA: (HBV DNA + HBV RNA) to assess the reverse transcriptional efficiency of pgRNA (f). Abbreviations: IT: immune-tolerant phase; IC: immune-clearance phases; LR: low-replicated phase; HBV: hepatitis B virus; HBsAg: hepatitis B surface antigen; HBcrAg: hepatitis B core-related antigen; cccDNA: covalently closed circular DNA; n: number of cases; ∗∗∗∗*P* < 0.0001; ∗∗∗0.0001 < *P* < 0.001; ∗∗0.001 < *P* < 0.01; ∗0.01 < *P* < 0.05; ns, *P* > 0.05.

The cccDNA transcription efficiency, represented by the ratio of serum HBV RNA to intrahepatic cccDNA (serum HBV RNA/cccDNA), was higher in HBeAg-positive patients (IT and IC) than in HBeAg-negative patients (LR and Reactivation) (1.02 *vs.* 0.30, *P* < 0.001, Figure 3). The same was true if the numerator of this ratio is not serum HBV RNA but serum HBV DNA and HBcrAg (*P* < 0.001). Unexpectedly, HBeAg-positive patients had lower ratio of serum HBsAg to intrahepatic cccDNA than HBeAg-negative patients (0.73 *vs.* 0.83, *P* < 0.001, Figure 3). The reverse transcription efficiency of HBV pgRNA was defined as the ratio of serum HBV DNA to the sum of HBV DNA and HBV RNA as we previously suggested [23]. HBeAg-positive patients had lower HBV pgRNA reverse transcription efficiency, compared with HBeAg-negative patients. The pgRNA reverse transcription efficiency median in this study was 0.56, 0.56, 0.70, and 0.67, for IT, IC, LR, and reactivation patients, respectively.

### 3.5. The Intrahepatic and Serological HBV Markers with Liver Inflammation

**Figure 4:**
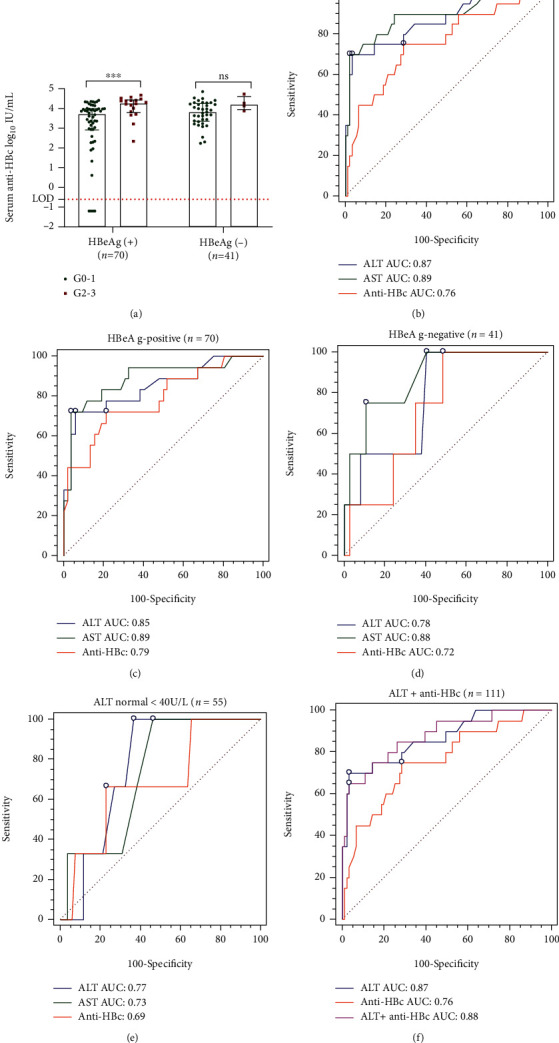
The receiver-operating characteristic curve (ROC) of serum anti-HBc was used to distinguish between mild (G0-1) and moderate to severe (G2-3) liver tissue inflammation. Abbreviations: ALT: alanine aminotransferase; AST: aspartate aminotransferase; HBV: hepatitis B virus; HBeAg: hepatitis B e antigen; anti-HBc: hepatitis B core antibody; n: number of cases; AUC: area under the ROC curve.

**Figure 5:**
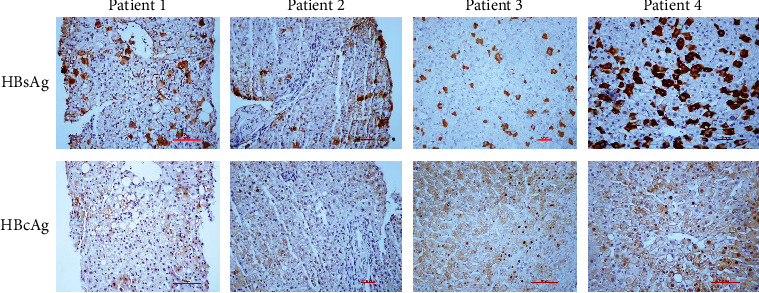
Immunohistochemical staining of HBsAg and HBcAg in the four anti-HBc-negative patients. Abbreviations: HBsAg: hepatitis B surface antigen; HBcAg: hepatitis B core antigen.

Serum HBsAg, HBeAg, HBV RNA, HBV DNA, HBcrAg, and liver HBV tDNA and HBV cccDNA levels, as well as anti-HBc, were analyzed for their association with liver histological inflammation (Metavir score grades). Univariate and multivariate analyses showed that in addition to biochemical indicators such as ALT and AST, serum anti-HBc level was the only marker showed a correlation with liver inflammation (Table 2). This result was expected as anti-HBc levels were reported to partly indicate host immune response to HBV infection. Further subgroup analysis in the HBeAg-positive patients revealed that patients with moderate to severe liver histological inflammation had higher anti-HBc level than patients with mild or without liver inflammation [4.24 (3.71, 4.41) *vs.* 3.65 (2.93, 3.99) log_10_ IU/mL, *P* < 0.01] (Figure 4). However, such difference disappeared between the ≥G2 and the ≤G1 groups [4.06 (3.27, 4.58) log_10_ IU/mL in ≥G2 group *vs.* 3.89 (3.39, 4.25) log_10_ IU/mL in ≤G1 group, *P* = 0.627] in the HBeAg-negative patients (Figure 4). Four patients in IT phase had undetectable anti-HBc levels and their liver inflammatory scores were all ≤ G1. The clinical information of these four patients is shown in Supplementary Table 1 and their HBsAg and HBcAg immunohistochemistry is shown in Figure 5.

Lastly, receiver-operating characteristic curve (ROC) assessment of the potential use of serum anti-HBc to distinguish patients with and without liver inflammation (≤G1 or ≥ G2). As shown in Figure 4, the area under the ROC curve (AUC) (95% CI) was 0.76 (0.67, 0.83), and the sensitivity and specificity were 75.00% and 71.43%, when taken a 4.00 log_10_ IU/mL serum anti-HBc cutoff according to the Youden index. The AUC increased to 0.79 (0.67, 0.88) in HBeAg-positive patients, with sensitivity as 72.22% and specificity as 78.85%; the AUC decreased to 0.72 (0.56, 0.85) in HBeAg-negative patients (sensitivity: 75.00%, specificity: 62.16%). At the cutoff value of 40 IU/L, the AUC (95% CI) of ALT was as high as 0.87 (0.76, 0.89) in all patients (sensitivity: 85.00%, specificity: 57.14%). The combined AUC (95% CI) for ALT plus anti-HBc levels was 0.88 (0.80, 0.93), with same sensitivity (85.00%) but improved specificity (73.63%), as compared to that of ALT alone (Figure 4).

## 4. Discussion

In this cross-sectional study, the dynamic changes of serum anti-HBc, HBV RNA, and HBcrAg levels, the classic markers HBsAg, HBV DNA, and ALT levels, and intrahepatic cccDNA levels across the distinct disease phases were characterized in treatment-naïve chronic HBV-infected patients. To our knowledge, few studies have comprehensively investigated all these serum and intrahepatic HBV markers, mainly restricted by obtaining biopsies from patients across distinct phases.

Consistent with the previous report [13], our results here in this study also showed that serum HBV RNA and HBcrAg levels were significantly higher in the IT- and IC-phase patients than in the LR- and reactivation-phase patients. Perhaps due to the reduced amount of cccDNA and suppressed transcriptional activity in HBeAg-negative patients [24, 25], the detection rate of serum HBV RNA and HBcrAg in HBeAg-negative patients were 26.83% (11 patients) and 53.66% (22 patients), respectively. Therefore, considering the low detection rates and levels of HBV RNA and HBcrAg, HBV DNA should be compared to monitor HBeAg-negative CHB in clinical practice. Consistent with the previous report [26], a good correlation between serum HBcrAg level and levels of HBV DNA and HBV RNA was observed in our analysis, higher than their correlation with other serum and intrahepatic HBV markers involved in the analysis.

We further verified that serum HBV RNA and HBcrAg levels could reflect intrahepatic cccDNA levels in patients at both HBeAg-positive and HBeAg-negative phases. In a total of 111 patients, the Spearman correlation coefficients (*r*) between intrahepatic cccDNA and serum HBV RNA, HBcrAg, and classical serum HBV DNA and HBsAg were 0.79, 0.78, 0.81, and 0.61, respectively. Comparatively, the correlation between HBsAg and other HBV markers involved in the analysis was relatively weak (*r* ≤0.70), especially for LR- and reactivation-phase patients. A potential explanation could be that serum HBV RNA and HBcrAg were only derived from cccDNA; in contrast, HBsAg could be derived from either cccDNA or integrated HBV DNA fragments [27]. Therefore, serum HBV RNA and HBcrAg levels may more accurately indicate intrahepatic cccDNA levels, and this makes them more potential to serve as surrogate markers for intrahepatic cccDNA. However, the correlation coefficients between various serum markers and cccDNA from strong to weak was HBV DNA > HBV RNA ≈ HBcrAg > HBeAg > HBsAg. Thus, unlike in antiviral-treated patients, serum HBV RNA and HBcrAg levels seem not superior to serum HBV DNA levels in reflecting cccDNA level in treatment-naïve patients.

In addition, the study here also found that HBeAg-positive patients (IT and IC) showed a higher transcriptional efficiency of cccDNA than HBeAg-negative patients (LR and Reactivation). The same was true if the numerator of this ratio is not serum HBV RNA but serum HBV DNA and HBcrAg. HBeAg seroconversion may reduce cccDNA transcription efficiency by epigenetic silence and cccDNA's mutation accumulation. However, HBeAg-positive patients showed a lower ratio of serum HBsAg to intrahepatic cccDNA than that in HBeAg-negative patients. Integrated HBV DNA may contribute to infection persistence by maintaining the liver expression of HBsAg, without generation of HBV RNA, HBcrAg, and HBV DNA from cccDNA [28]. Furthermore, the data here demonstrated that unlike the inferior reverse transcription efficiency in HBeAg-positive patients, HBeAg-negative patients could maintain a more efficient reverse transcription to turn pgRNA into rcDNA, providing evidence that HBV DNA is more easily detected than HBV RNA in LR- and reactivation-phase patients.

As an indicator of host immune response to HBV infection, the pattern of serum anti-HBc level was quite different from those of serum HBV DNA, HBV RNA, and HBcrAg levels across distinct phases. The results of the study showed that the serum anti-HBc level of the IT-phase patients was lower than that of the other three phase patients, and only IT-phase patient's serum anti-HBc levels and cccDNA levels had a negative correlation. These results implicated presence of active anti-HBV-specific immune response even in patients in the IT phase, and support that serum anti-HBc levels are closely associated with both HBV replication and host immune response [29]. Another supporting evidence is that four IT-phase patients were found with undetectable anti-HBc and had a high viral replication but with non to mild histological liver inflammation. It is speculated that a high viral load concomitant with a large number of naked capsid particles may exhaust the low levels of anti-HBc produced in patients in the IT phase [30].

Serum HBV RNA level has been previously reported to reflect liver histological changes in NAs-treated CHB patients [8]. In this study, serum anti-HBc was found to be the only marker associated with liver inflammation by univariate and multivariate analyses. In the HBeAg-positive patients, patients with moderate/severe liver histological changes had significantly higher serum anti-HBc levels than those with no/mild liver histological changes. In all patients, although the AUC to indicate liver inflammation by serum anti-HBc level was lower than that by ALT level (0.76 *vs.* 0.87), the combination of the two greatly improved the specificity (57.14% *vs.* 73.63%). Notably, serum anti-HBc levels with a cutoff value of 4.00 log_10_ IU/mL to indicate liver inflammation had a higher specificity in HBeAg-positive patients (78.85%) than in HBeAg-negative patients (62.16%), suggesting that serum anti-HBc level can better mirror hepatic inflammation in HBeAg-positive patients than in HBeAg-negative patients. Therefore, serum anti-HBc level can compensate for the weak specificity of ALT in distinguishing HBV-related liver inflammation.

The study has limitations. The sample size, especially for the reactivation-phase group, was relatively small (12 patients). In addition, limited liver biopsies from HBeAg-negative patients with moderate/severe liver inflammation were obtained. All of these result in reduced statistical power.

**Figure 6:**
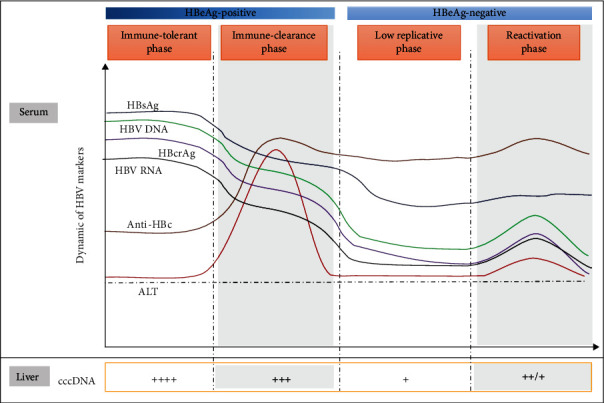
Schematic diagram of HBV serum markers and intrahepatic cccDNA levels across the distinct phases of chronic HBV infection. Abbreviations: cccDNA: covalently closed circular DNA; HBV: hepatitis B virus; HBeAg: hepatitis B e antigen; HBsAg: hepatitis B surface antigen; HBcrAg: hepatitis B core-related antigen; anti-HBc: hepatitis B core antibody; tDNA: total DNA.

In summary, we provided a comprehensive picture of all these serological markers in untreated patients across the distinct phases of HBV chronic infection (Figure 6). Serum HBV RNA and HBcrAg levels could mirror intrahepatic cccDNA levels across the distinct phases in treatment-naïve CHB patients, but the two serum markers seemed not superior to serum HBV DNA. Other major findings were that serum anti-HBc level was negatively linked with cccDNA level in IT phase rather than the other three phases; and serum anti-HBc level could better mirror liver inflammation in HBeAg-positive patients than in HBeAg-negative patients. These findings further our understanding of the clinical values of the novel serum HBV markers that may help the management of CHB.

## Figures and Tables

**Table 1:** Clinical and virological details of the treatment-naïve HBV-infected patients.

Characteristic	HBeAg-positive patients (*n* =70)			HBeAg-negative patients (*n* =41)		
	Immune-tolerant phase (*n* =21)	Immune-clearance phase (*n* =49)	*P*-value	Low replicative phase (*n* =29)	Reactivation phase (*n* =12)	*P*-value
Gender, male, *n* (%)	7 (33.33)	32 (65.31)	0.027	18 (62.07)	11 (91.67)	0.073
Age, years	38 (33, 45)	32 (27, 38)	0.007	38 (31, 42)	38 (30, 44)	0.682
HBV DNA, log_10_ IU/mL	8.57 (8.12, 9.00)	8.52 (7.59, 9.00)	0.636	2.37 (1.94, 3.04)	4.27 (3.44, 5.08)	< 0.001
HBsAg, IU/mL	52010 (36221, 77630)	27173 (5731, 49565)	0.009	1516 (231, 4814)	1176 (608, 7741)	0.921
HBeAg, COI	1710 (1447, 1964)	1674 (853, 1842)	0.231	/	/	/
ALT, U/L	25 (19, 33)	76 (49, 243)	< 0.001	19 (17, 28)	54 (39, 79)	< 0.001
AST, U/L	25 (19, 30)	42 (31, 100)	< 0.001	21 (18, 25)	33 (25, 51)	< 0.001
ALB, g/L	39 (38, 43)	41 (37, 44)	0.714	43 (40, 45)	44 (40, 45)	0.854
TBIL, *μ*mol/L	12.60 (9.50, 14.90)	14.70 (11.50, 19.20)	0.040	11.20 (8.70, 16.70)	16.60 (10.50, 25.00)	0.088
*γ*-GT	19.50 (13.50, 23.00)	30.00 (18.00, 55.50)	0.010	20.00 (16.00, 29.00)	25.50 (21.50, 47.30)	0.015

Abbreviations: ALT: alanine aminotransferase; AST: aspartate aminotransferase; ALB: albumin; HBV: hepatitis B virus; HBeAg: hepatitis B e antigen; HBsAg: hepatitis B surface antigen; n: number of cases; TBIL: total bilirubin; *γ*-GT: *γ*-glutamyltransferase. All the results of continuous variable are described as median (interquartile range).

**Table 2:** Univariate and multivariate logistic regression analysis of liver inflammation related indicators.

	METAVIR, inflammatory activity (grading)		Univariable	Multivariable	
	Grading score< 2 (*n* = 91)	Grading score ≥ 2 (*n* = 20)	*P-*value	OR (95% CI)	*P*-value
Gender, male, *n* (%)	57 (62.64)	11 (55.00)	0.703		
Age, years	35 (29, 42)	35 (27, 45)	0.905		
ALT, U/L	33 (20, 55)	278 (57, 657)	< 0.001	1.008 (0.997, 1.020)	0.154
AST, U/L	27 (21, 34)	113 (45, 440)	< 0.001	1.004 (0.986, 1.022)	0.685
HBsAg, IU/mL	15299 (1439, 49212)	6587 (1709, 22416)	0.234		
HBeAg, COI	988.70 (0.11, 1786)	691.00 (9.78, 1482.50)	0.851		
HBV DNA, log_10_ IU/mL	7.46 (3.04, 9.00)	7.72 (6.48, 8.78)	0.283		
HBV RNA, copies/mL	5.69 (1, 6.82)	6.03 (4.36, 6.40)	0.504		
HBcrAg, log_10_ U/mL	8.21 (3.04, 8.91)	8.04 (6.70, 8.50)	0.676		
Anti-HBc, log_10_ IU/mL	3.82 (3.15, 4.16)	4.27 (3.88, 4.46)	< 0.001	2.231 (0.605, 5.19)	0.273
Intrahepatic tDNA, log_10_ copies/cells	7.42 (4.99, 7.98)	7.03 (6.29, 7.37)	0.878		
Intrahepatic cccDNA, log_10_ copies/cells	5.47 (3.85, 6.52)	5.59 (5.02, 6.24)	0.857		

Abbreviations: ALT: alanine aminotransferase; AST: aspartate aminotransferase; cccDNA: covalently closed circular DNA; HBV: hepatitis B virus; HBeAg: hepatitis B e antigen; HBsAg: hepatitis B surface antigen; HBcrAg: hepatitis B core-related antigen; Anti-HBc: hepatitis B core antibody; tDNA: total DNA.

## Data Availability

The authors confirm that the data supporting the findings of this study are available on the request from the corresponding author.

## Supplementary Materials

Supplementary Materials Supplementary Table 1. Clinical and virological details of the four anti-HBc-negative individuals.
