# Supplementary material for: Characteristics of HBV Novel Serum Markers across Distinct Phases in Treatment-Naïve Chronic HBV-Infected Patients
Source: Dis Markers. 2022 Jul 14;2022:4133283. doi: 10.1155/2022/4133283 (PMC9303147; doi:10.1155/2022/4133283)
Supplement: Supplementary Materials — Supplementary Table 1. Clinical and virological details of the four anti-HBc-negative individuals. [file 4133283.f1.docx]

Supplementary Table 1 Clinical and virological details of the four Anti-HBc-negtive individuals

| Patient ID | Gender | Age, years | ALT, U/L | AST, U/L | HBsAg, IU/mL | Anti-HBs, IU/L | HBeAg, COI | HBV DNA, log_10_ IU/mL | HBV RNA, log_10_ copies/mL | HBcrAg, log_10_ U/mL | Anti-HBc, IU/mL | Intrahepatic HBV tDNA log_10_ copies/10^5^ cell | Intrahepatic HBV cccDNA log_10_ copies/10^5^ cell |
| --- | --- | --- | --- | --- | --- | --- | --- | --- | --- | --- | --- | --- | --- |
| patient No.1 | Female | 43 | 38 | 17.5 | 78760 | <2 | 2056 | 8.54 | 6.90 | 8.953 | < 0.25 | 7.66 | 6.99 |
| patient No.2 | Female | 38 | 38 | 8.4 | 156790 | 30.73 | 1981 | 8.35 | 6.87 | 9.196 | < 0.25 | 7.79 | 6.85 |
| patient No.3 | Female | 32 | 37 | 6.5 | 70680 | <2 | 1992 | 8.57 | 7.21 | 9.074 | < 0.25 | 7.63 | 6.32 |
| patient No.4 | Female | 44 | 36 | 11.8 | 88900 | <2 | 2003 | 9.00 | 6.62 | \ | < 0.25 | 7.73 | 6.72 |

Abbreviations: ALT, alanine aminotransferase; AST, aspartate aminotransferase; cccDNA, covalently closed circular DNA; HBV, hepatitis B virus; HBeAg, hepatitis B e antigen; HBsAg, hepatitis B surface antigen; HBcrAg, hepatitis B core-related antigen; Anti-HBc, hepatitis B core antibody; Anti-HBs, hepatitis B surface antibody; tDNA, total DNA.
